# Fertility treatment and oral contraceptive discontinuation for identification of pregnancy planning in routinely collected health data – an application to analgesic and antibiotic utilisation

**DOI:** 10.1186/s12884-020-03435-4

**Published:** 2020-11-25

**Authors:** Sarah Hjorth, Mollie Wood, Fatima Tauqeer, Hedvig Nordeng

**Affiliations:** 1 PharmacoEpidemiology and Drug Safety Research Group, Department of Pharmacy, and PharmaTox Strategic Initiative, Faculty of Mathematics and Natural Sciences, University of Oslo, Postboks 1068 Blindern, 0316 Oslo, Norway; 2 Department of Epidemiology, T.H. Chan School of Public Health, Harvard University, Boston, USA; 3 Department of Child Health and Development, Norwegian Institute of Public Health, Oslo, Norway

**Keywords:** “Analgesics, Non-narcotic”, “Analgesics, Opioids”, “Anti-bacterial agents”, “Drug Utilization”, “Pregnancy, Unplanned”, “Registries”

## Abstract

**Background:**

Women with unplanned pregnancies use folic acid less frequently, and more often use potentially teratogenic medications in the first trimester. Yet most studies based on routinely collected data lack information on pregnancy planning. Further, only pregnancies proceeding beyond a certain gestational age appear in routinely collected data, creating the possibility for collider-stratification bias. If pregnancy intention could be identified, pregnancies could be ascertained earlier. This study aimed to investigate fertility treatment and discontinuation of oral contraception (OC) as proxies for pregnancy planning by describing variations in patterns of prescription fills for antibiotics and analgesics during the peri-pregnancy period by these proxies of pregnancy intention.

**Methods:**

Fertility treatment with clomiphene and discontinuation of OC were identified in the Norwegian Prescription Database (NorPD) and linked with data from the Medical Birth Registry of Norway for the years 2006 to 2017. Filled prescriptions for antibiotics and analgesics from NorPD were displayed for women on fertility treatment, women who discontinued OC before pregnancy, and women who discontinued during pregnancy.

**Results:**

Of 172,585 included pregnancies, fertility treatment was identified in 19,449, and OC discontinuation before or during pregnancy in 153,136. Women who discontinued OC during pregnancy were less likely to use preconception folic acid (25.4%) than women who discontinued before pregnancy (32.9%), and women on fertility treatment (51.0%). Proportions of first trimester prescription fills were 4.9% (analgesics) and 12.8% (antibiotics) for women who discontinued OC during pregnancy, compared to 4.0 and 11.4% in women who discontinued OC before pregnancy, and 4.7 and 11.0% in women on fertility treatment.

**Conclusions:**

There were no substantial differences in patterns of prescription fills for analgesics and antibiotics before or during pregnancy by fertility treatment and OC discontinuation. This suggests that there were few differences in medication use between women with planned and unplanned pregnancies, or that fertility treatment and timing of OC discontinuation from routinely collected health data cannot stand alone in the identification of unplanned pregnancies. A narrower definition of OC discontinuation during pregnancy seemed to be a better proxy, but this should be confirmed in other studies.

**Supplementary Information:**

The online version contains supplementary material available at 10.1186/s12884-020-03435-4.

## Background

An estimated 45% of pregnancies in high-income countries and 29% of pregnancies in Northern Europe are unplanned [1]. Women with unplanned pregnancies are less likely to use folic acid before pregnancy [2, 3], more likely to smoke [2, 4], and more often use medications in the first trimester, including potentially teratogenic medications [5]. Most studies on pregnancy intention and lifestyle in pregnancy have used self-reported data [3]. In routinely collected health data, where study samples are more representative of the underlying population, information on pregnancy intention is typically unavailable. Therefore, studies of prescription drug utilisation or safety usually lack information on pregnancy intention [6–8]. Further, for pregnancies to appear in routinely collected data and be identified in studies, they need to proceed beyond a certain point, for example week 12 [7], or end in live births [6]. Hence, early pregnancy losses cannot be identified, and collider-stratification bias may be introduced [9]. If pregnancy intention could be identified in routinely collected data, it would be possible to identify pregnancies prospectively from an earlier time.

A way to study pregnancy intention in routinely collected data could be to identify women on fertility treatment. A previous study investigated prescription drug utilisation in women on fertility treatment compared to women with spontaneous pregnancies [10]. However, to make inferences about women with planned pregnancies who conceive spontaneously, a different approach is needed. A few studies on pregnancy intention and lifestyle in pregnancy have used oral contraceptive discontinuation as a marker of pregnancy intention [3]. To our knowledge, this approach has not been used in drug utilisation studies. Folic acid use may also be a marker of pregnancy planning in data sources that record such use, as most public health authorities recommend 400 micrograms folic acid daily from at least 1 month prior to conception and throughout the first 12 weeks of pregnancy [11]. We therefore aimed to investigate fertility treatment and discontinuation of oral contraception as proxies for pregnancy planning by describing variations in patterns of prescription fills for antibiotics and analgesics during the peri-pregnancy period by these proxies of pregnancy intention, while accounting for folic acid use.

Antibiotic and analgesics were chosen for two reasons. First, they are commonly used classes of prescription medications in pregnancy [6–8]. In a US population, prescription antibiotics were filled in 50%, and analgesics in 30% of pregnancies [6]. Second, we expected that the two classes would show different patterns of use in the peri-pregnancy period. Previous studies have found that prescription analgesic use declines over the course of pregnancy, whereas prescription antibiotic use does not [7]. In studies based on self-reported surveys, women with unplanned pregnancies had 29% higher odds of reporting use of prescription medications for chronic or long-term illnesses [12], and 54% higher likelihood of reporting frequent paracetamol use in the first trimester [13]. There was no difference in the use of prescription medications for acute indications, such as urinary tract infections [12]. However, no studies have replicated these results in a population-based sample. Furthermore, medication use was studied either as any use during pregnancy [12], or in the first trimester only [13], and it is unknown whether pregnancy intention will affect medication use beyond the first trimester.

We sought to answer these lingering questions, using a population-based cohort of Norwegian women. Specifically, we evaluated fertility treatment or discontinuation of oral contraceptives (OC) as proxies for pregnancy planning, with the hypothesis that 1) women on fertility treatment, and women who discontinue OC before pregnancy, would be less likely to use prescription analgesics before pregnancy and in the first trimester than women who discontinue OC during pregnancy, and 2) there would be no difference in the use of prescription antibiotics. Additionally, we expected that in the second and third trimester, and after pregnancy, there would be no difference in the use of prescription analgesics and antibiotics by proxies for pregnancy planning.

## Methods

### Study population and data sources

The study was based on data from the Norwegian Prescription Database (NorPD) and the Medical Birth Registry of Norway (MBRN), which were linked using unique personal identification numbers. NorPD was established in 2004 and contains information on all prescriptions filled at pharmacies [14]. Prescriptions are classified using the World Health Organization’s Anatomical Therapeutic Chemical (ATC) Classification System [15]. The MBRN was established in 1967 [16] and contains data on all pregnancies with a duration beyond week 12 [17]. The MBRN has information on the date of birth of the child and the gestational age at birth, allowing calculation of the start of pregnancy.

**Fig. 1:**
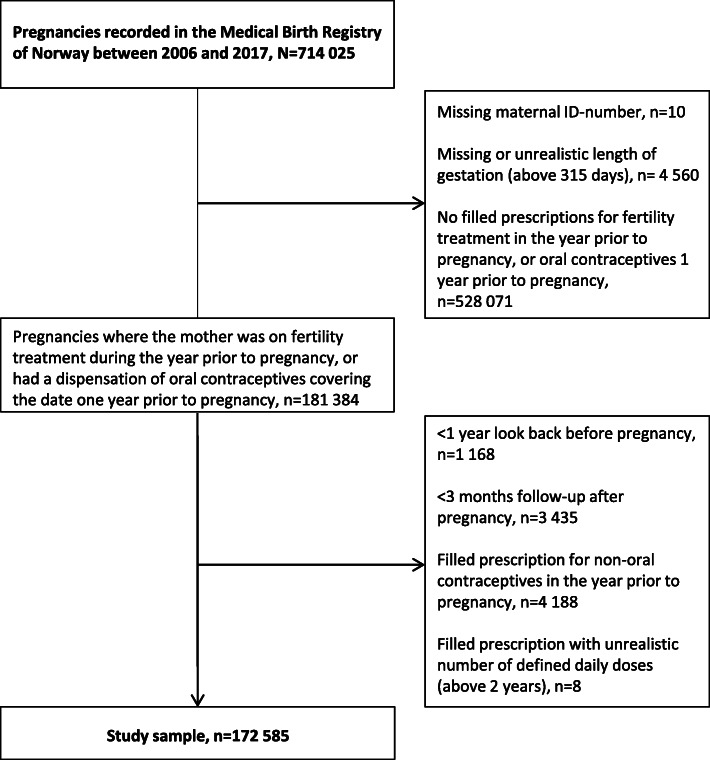
Flowchart of the study sample

We included all registered pregnancies from 2006 to 2017. To make inferences about pregnancy intention, we included women with either filled prescriptions for medications for fertility treatment in the year before pregnancy, or OC dispensed that covered the date 1 year before the start of pregnancy. We focused on OC to be able to approximate the timing of contraceptive discontinuation; women using alternate forms of long-acting birth control (e.g., intrauterine devices) were excluded from our sample. Figure 1 shows the exclusion criteria applied to achieve the study sample.

### Variables

#### Proxies for pregnancy intention

**Fig. 2:**
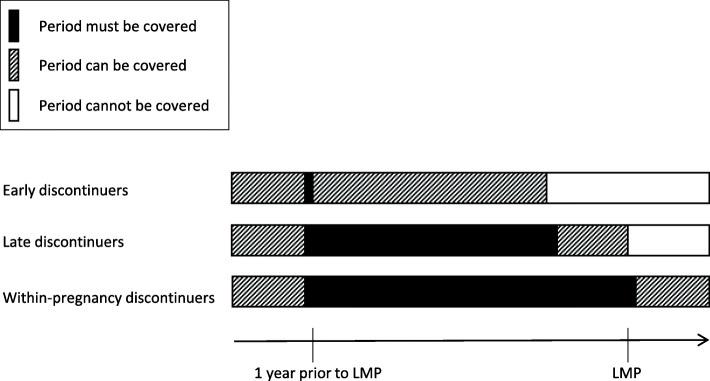
Timing of discontinuation in the three oral contraceptive discontinuation groups. LMP: Start of pregnancy

Fertility treatment was identified in NorPD as filled prescriptions for clomiphene (ATC-code G03GB02) in the year before pregnancy. Clomiphene is the first-line medication in fertility treatment in Norway [18]. OC use was assessed by filled prescriptions for hormonal contraception for systemic use (ATC-code G03A, excluding contraceptive patches, G03AA13, injections, G03AC06, implants, G03AC08, and emergency contraceptives, G03AD). Discontinuation status was divided into three categories: Early OC discontinuers, late OC discontinuers, and within-pregnancy OC discontinuers (Fig. 2). Early OC discontinuers had their last day covered by OCs between 1 year before pregnancy and three cycles before pregnancy, where each cycle was assumed to last 28 days. Late OC discontinuers had their last day covered between two cycles and 27 days before pregnancy and 1 day before pregnancy. Within-pregnancy OC discontinuers had coverage overlapping with pregnancy.

To account for folic acid use, we stratified the groups by timing of folic acid initiation: initiation before pregnancy, during pregnancy, or no folic acid. Information on folic acid initiation was obtained from MBRN (recorded by health care professionals) and/or NorPD (filled prescriptions of ATC-codes B03BB, A11EA or A11EB).

The validity of data on fertility treatment with clomiphene and OC discontinuation has not been investigated for NorPD. A Finnish study found that 10% of pregnancies were misclassified as spontaneous if fertility treatment was identified by prescription fills [19]. A US study comparing claims data to self-report of OC use found moderate agreement, Cohen’s kappa 0.46 [20].

A study investigating the validity of preconception folic acid registration in MBRN found underreporting: 45% of self-reported users before pregnancy were registered as non-users. The validity was not investigated for registration of folic acid use during pregnancy [21].

#### Drug utilisation

We studied five peri-pregnancy periods: 90 days before pregnancy, first trimester, second trimester, third trimester, and 90 days after pregnancy. For each period, we identified the proportion of women who filled a prescription for an analgesic (ATC-code N02 or M01A) or an antibiotic (ATC-code J01) from NorPD. For antibiotics, we included prescriptions where the defined daily doses overlapped with the period, because antibiotics are used continuously. In addition, we studied prescription fills for non-steroidal anti-inflammatory drugs (NSAIDs, ATC-code M01A) and tetracyclines (ATC-code J01A) separately, as these medications are contraindicated in the second half of pregnancy in Norway [22, 23].

#### Participant characteristics

We included information from MBRN on sociodemographics, lifestyle, and obstetric factors including obstetric comorbidity index, adapted from Bateman et al. [24].

### Statistical analysis

Participant characteristics by proxies for pregnancy planning were compared using descriptive statistics. For prescription fills, we compared proportions with 95% confidence intervals (CIs). The proportion of missingness was compared for variables with missing values.

In a sensitivity analysis to account for uncertainty in date of conception, the start of pregnancy was considered as start of pregnancy 1) plus 14 days, and 2) minus 14 days. In another sensitivity analysis to improve specificity, we considered only women with filled prescriptions for OC one cycle before pregnancy as within-pregnancy discontinuers. A third sensitivity analysis included women with filled prescriptions of vaginal rings with hormones (ATC-code G02BB) and/or contraceptive patches. A fourth sensitivity analysis was restricted to live births, as claims data often only is reliable for live births [25]. All analyses were performed using Stata (version16;StataCorpLP).

## Results

Of 172,585 included pregnancies, 19,449 occurred after fertility treatment with clomiphene. Among 153,136 women with OC coverage 1 year before pregnancy, 50.8% were early, 27.8% late, and 21.4% within-pregnancy discontinuers. Of women on fertility treatment, half (51.0%) initiated folic acid use before pregnancy. The proportion of women who initiated folic acid before pregnancy was lower for early (35.3%), late (32.9%), and within-pregnancy OC discontinuers (25.4%).

Early OC discontinuers were more likely to have experienced previous pregnancy loss than within-pregnancy OC discontinuers (Table 1). Women on fertility treatment were older, had more obstetric comorbidities, and were more likely to be nulliparous and to have experienced previous pregnancy loss. Women with no folic acid use were more likely to smoke in early and late pregnancy (Additional file 1).

**Table 1:** Characteristics of the included pregnancies by proxies of pregnancy intention^a,b^

	Fertility treatment (*n* = 19,449)	Timing of oral contraceptive discontinuation		
		Early (*n* = 77,735)	Late (*n* = 42,621)	Within-pregnancy (*n* = 32,780)
Maternal age	31.8 (5.0)	28.9 (4.7)	28.4 (4.6)	27.7 (4.9)
Married/cohabiting	95.3	94.4	94.9	92.4
Employed	73.2	74.1	75.5	72.1
Nulliparous	58.2	55.3	57.6	59.4
Previous pregnancy loss	27.5	19.9	10.8	9.9
Obstetric comorbidity index^c^	0.77 (1.2)	0.39 (0.8)	0.36 (0.8)	0.35 (0.9)
*Components of the index*				
Asthma	5.3	5.2	5.3	5.4
Diabetes, pre-gestational	1.2	0.7	0.7	0.7
Hypertension, chronic	0.9	0.5	0.4	0.4
Hypertension, gestational	2.5	2.1	2.1	1.9
Kidney disease	0.6	0.7	0.7	0.6
Multiple gestation	5.6	1.3	1.3	1.2
Preeclampsia, mild	3.0	2.0	2.1	2.1
Preeclampsia, severe	2.1	1.2	1.3	1.6
Previous caesarean section	5.5	5.3	4.6	3.6
Rheumatoid arthritis	0.7	0.4	0.4	0.3
Folic acid use				
Initiation before pregnancy	51.0	35.3	32.9	25.4
Initiation during pregnancy	31.4	43.4	44.9	49.1
No folic acid	17.7	21.6	22.2	25.5
Smoking in early pregnancy	5.4	8.1	7.9	9.9
Smoking at the end of pregnancy	3.3	4.6	4.5	5.4
Weight gain in pregnancy	13.8 (7.7)	14.6 (8.5)	14.6 (7.3)	14.9 (7.9)

^a^ Table stratified by folic acid use is shown in Additional file 1

^b^Figures shown are percent of non-missing values with the exception of maternal age, calendar year, obstetric comorbidity index, and weight gain in pregnancy, presented as mean (standard deviation). Missing values ranged from 0% (maternal age, calendar year, parity) to 17.6% (maternal employment). Women could choose not to have smoking and weight reported to the MBRN. For smoking, 12.4 to 16.3% chose not to report. For weight, 75.9 to 77.7% chose not to report

^c^Adapted from Bateman et al. [24], using the variables available in MBRN (age, asthma, pre-gestational diabetes, chronic hypertension, kidney disease, previous caesarean section, multiple gestation, severe preeclampsia, mild preeclampsia, gestational hypertension) and weighting the variables as done by Bateman et al. [24].

### Analgesics

In total, 9.8% filled a prescription for an analgesic before pregnancy. In all groups, proportions declined in the first trimester, continued to decline throughout pregnancy, and rose after pregnancy, but not to pre-pregnancy levels.

**Fig. 3:**
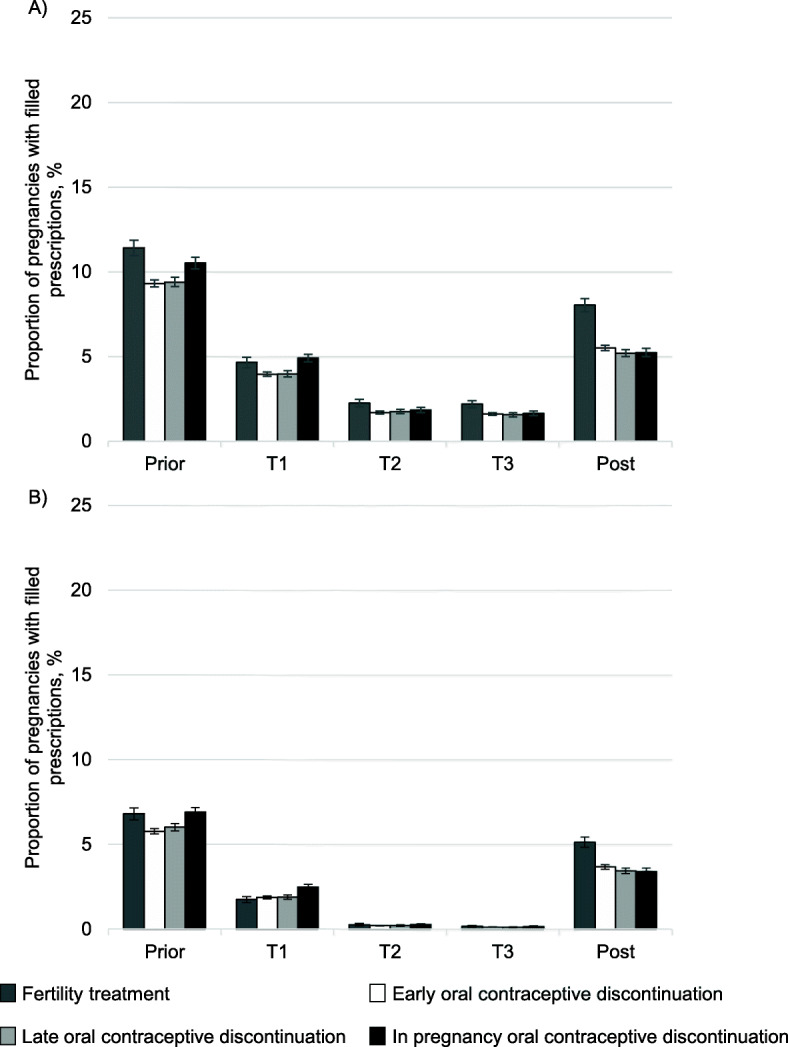
Proportion of pregnancies with analgesic prescription fills by peri-pregnancy period and proxies of pregnancy intention. Panel **a**: Any analgesics, panel **b**: non-steroidal anti-inflammatory drugs. T: Trimester. Error bars represent the 95% confidence intervals. Unstratified on folic acid for clarity of interpretation. A stratified table is shown in Additional file 2

Within-pregnancy OC discontinuers had similar proportions of analgesic prescription fills before pregnancy (10.5, 95% CI 10.2 to10.9%) to early (9.3, 95% CI 9.1 to 9.5%) and late discontinuers (9.4, 95% CI 9.1 to 9.7%) (Fig. 3, panel a). Women on fertility treatment filled the most prescriptions for analgesics (11.4, 95% CI 11.0 to 11.9%). During pregnancy, there were no substantial differences by group. After pregnancy, there were no differences in analgesic prescription fills by OC group, but women in the fertility treatment group had higher proportions of analgesic prescription fills (8.0, 95% CI 7.7 to 8.4%) than OC discontinuers (5.2 to 5.5%, 95% CIs 5.0 to 5.4% and 5.4 to 5.7%, respectively).

Prescription fills for NSAIDs followed the same patterns across peri-pregnancy periods and proxies for pregnancy planning as analgesics overall (Fig. 3, panel b).

### Antibiotics

For all groups, the proportion of filled antibiotic prescriptions was similar before (12.0%), and during (12.4%) pregnancy. After pregnancy, the proportion rose (18.4%).

**Fig. 4:**
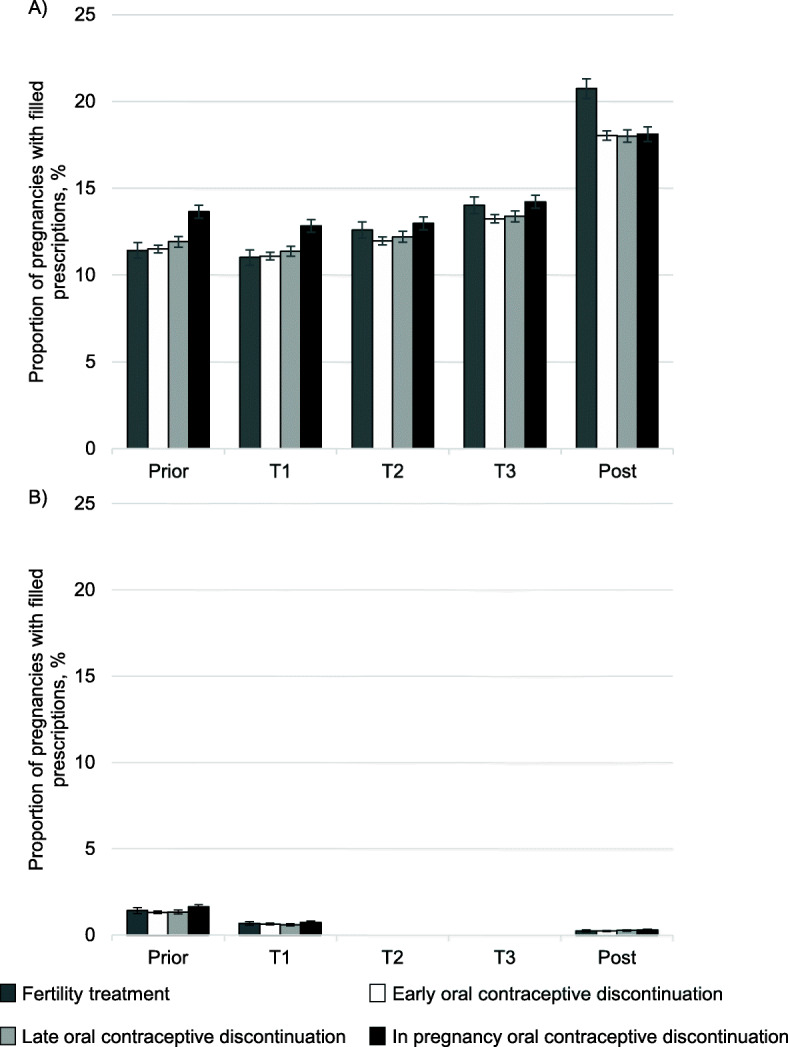
Proportion of pregnancies with antibiotic prescription fills by peri-pregnancy period and proxies of pregnancy intention. Panel **a**: Any antibiotics, panel **b**: tetracyclines. T: Trimester. Error bars represent the 95% confidence intervals. For tetracyclines, results are not shown for T2 and T3 for reasons of confidentiality, as less than five pregnancies were exposed in some of the groups. Unstratified on folic acid for clarity of interpretation. A stratified table is shown in Additional file 3

Within-pregnancy OC discontinuers had the highest proportion of antibiotic prescription fills before pregnancy (13.7, 95% CI 13.3 to 14.0%), but the proportions were quite similar for early (11.5, 95% CI 11.3 to 11.7%) and late OC discontinuers (11.9, 95% CI 11.6 to 12.2%), and women on fertility treatment (11.4, 95% CI 11.0 to 11.9%) (Fig. 4, panel a), with comparable patterns in the first trimester. Antibiotic prescription fills did not differ by proxies for pregnancy intention in the second and third trimester. After pregnancy, women in the fertility treatment group filled more prescriptions for antibiotics (20.7, 95% CI 20.2 to 21.3%) than OC discontinuers (18.0 to 18.1%, 95% CIs 17.7 to 18.4% and 17.7 to 18.6%, respectively).

Prescription fills for tetracyclines decreased throughout pregnancy, and was never higher than 0.8% in any of the groups. The proportion rose slightly after pregnancy, but not to pre-pregnancy levels (Fig. 4, panel b). There were no differences in proportion of tetracycline fills by proxies for pregnancy intention.

### Sensitivity analyses

In different sensitivity analyses, we redefined pregnancy start as 14 days earlier or later, included women with filled prescriptions of vaginal rings with hormones and/or contraceptive patches, and restricted to live births. Participant characteristics by group and patterns of prescription fillings were largely unchanged (Additional files 4, 5, 6 and 7).

In women who filled prescriptions of OC within one cycle before pregnancy, 14.7% used folic acid before pregnancy. The women were slightly younger, less likely to be married, and more likely to smoke during pregnancy compared to within-pregnancy OC discontinuers in the primary analysis (Additional file 8). They also tended to have filled more analgesic and antibiotic prescriptions before pregnancy and in the first trimester than early and late OC discontinuers.

## Discussion

In this population-based cohort of 172,585 Norwegian pregnancies, we found no substantial differences in filled prescriptions for analgesics and antibiotics in the peri-pregnancy period by OC group. There were no substantial differences in prescription fills for the potentially harmful medication groups NSAIDs and tetracyclines either. This suggests that there were few differences in medication use between women with planned and unplanned pregnancies, or that fertility treatment and timing of OC discontinuation are not good proxies for pregnancy planning.

Women in the fertility treatment group were more likely to fill prescriptions for analgesics and antibiotics after pregnancy. In all groups, we found similar patterns across strata of folic acid use. Women who used folic acid prior to pregnancy had the lowest proportion of analgesic, and antibiotic prescription fills before pregnancy and in the first trimester. However, the proportions were not substantially different from those in women who used folic acid during pregnancy only, and women who did not use folic acid.

### Interpretation

A previous study found that women on fertility treatment were twice as likely to fill prescriptions for potentially hazardous medications in pregnancy as women with spontaneous pregnancies [10]. We did not find differences in prescription fills during pregnancy, but after pregnancy women in the fertility treatment group had the highest proportion of antibiotic and analgesic prescription fills. An explanation could be that women on fertility treatment are older and have more comorbidities.

The high proportions of filled prescriptions for antibiotics before pregnancy and in the first trimester in within-pregnancy OC discontinuers, especially those who filled a prescription within one cycle before pregnancy, are unlikely to be explained by reduced OC efficacy among women on antibiotics. One study found an association between any antibiotic prescription fills and reduced OC efficacy [26]. However, most studies found no association for systemic antibacterials (ATC group J01) [27, 28]. Differences in participant characteristics between our study and the previous volunteer-based studies that we based our hypotheses on, could explain the differences in findings. Participants in volunteer-based studies are often healthier and have a higher socioeconomic position than the general population. In our sample, included women were similar to the general birthing population in measured health parameters (Additional file 9).

We found that women with no folic acid use were more likely to smoke in early pregnancy (an expected marker of unplanned pregnancy) and in late pregnancy (not expected to indicate pregnancy intention), and less likely to be employed. A study from Norway found that self-reported pregnancy planning predicted folic acid use, but the strongest predictor was high education level [29]. Folic acid might, therefore, be a marker of both pregnancy intention and socioeconomic position.

### Limitations

A limitation of our chosen proxies for pregnancy planning is the inability to assess pregnancy planning in women who did not use contraception, or who used other types of contraception than short-acting hormonal. This meant that only 24.2% of the population could be classified by proxies for pregnancy intention. We did not include women without short-acting hormonal contraceptive use as a control group in our study, as they are likely a heterogeneous group of women who plan pregnancies, and women who do not plan pregnancies, but use other types of contraception. Furthermore, we ascertained that women were under observation for 1 year before the start of pregnancy by identifying prescription fills in NorPD. For women without contraceptive or fertility treatment use, 1 year of look-back would only be available if they had filled other prescriptions, thus introducing different selection criteria among the groups. Some conditions, including migraine with aura, hypertension, or a history of thromboembolic disorders, are considered contraindications for using hormonal contraceptives [30], and so women with these diagnoses are likely under-represented in our sample. We did not have information on migraine or thromboembolic disorders, but we found a similar prevalence of chronic hypertension in women on OC and women who were excluded from our study sample (Additional file 9). The main differences between included and excluded women were that included women were younger and more often nulliparous. Hence, our findings on patterns of prescription fills in peri-pregnancy may not be generalisable to the entire pregnant population. However, our findings regarding the selected proxies for pregnancy planning should be applicable to other settings that have the same availability of fertility treatment and similar use contraceptive medications.

We had no information on women who did not become pregnant, had early miscarriages, or terminations of pregnancy occurring before gestational week 12. Women with unplanned pregnancies may be more likely to have early terminations, leading to an underrepresentation of unplanned pregnancies in our sample. Women who have early terminations may differ systematically from women who continue pregnancy in lifestyle and medication use, so the underrepresentation could have attenuated group differences in our study. However, the absolute number of pregnancies missing from this study due to early terminations should be small. There are 13 early terminations per 1000 women in Norway, and in 19% of these, women report the use of short-acting hormonal contraceptives at pregnancy start [31].

Early miscarriages may be more likely among women with low fertility. One of our proposed markers for pregnancy planning - early OC discontinuation - may be a proxy for long time-to-pregnancy and therefore low fertility. We found that women on fertility treatment and early OC discontinuers were more likely to have experienced previous pregnancy loss. Women with pregnancy intention and long time-to-pregnancy may, therefore, be underrepresented in our study sample. Women with longer time-to-pregnancy will have had more time to make lifestyle changes before pregnancy; hence the underrepresentation could have attenuated group differences in our study. In cohorts that only have access to live births, underrepresentation of women with low fertility may be larger, as we found that women on fertility treatment were more often excluded in the sensitivity analysis restricted to live births. Repeating our study in a cohort that is not selected on achieving a pregnancy of at least 12 weeks’ duration may provide results that are more representative.

Misclassification of the proxies for pregnancy intention cannot be ruled out. We found that 21.4% of pregnancies between 2006 and 2017 were to women who filled a prescription for an OC in the year before pregnancy. A previous study showed that around 20% of Norwegian women of fertile age use OC [32], supporting the sensitivity of our classification. Women could have discontinued earlier than their last day covered, reducing specificity. This seems likely, as 21.4% of OC users were classified as within-pregnancy discontinuers. When we considered only women with filled OC prescriptions one cycle before pregnancy as within-pregnancy discontinuers to increase the specificity of within-pregnancy OC discontinuation, the proportion decreased from 21.4 to 4.7%. In addition, we saw lower proportions of folic acid use before pregnancy, and higher proportions of smoking and filled prescriptions in the first trimester, as expected. This classification might, therefore, warrant further investigation.

We identified fertility treatment with clomiphene in 2.7% of pregnancies between 2006 and 2017. Norwegian health authorities reported fertility treatment in 3 to 4% of pregnancies from 2003 to 2013 [33, 34]. It is possible that some women on fertility treatment were not treated with clomiphene. Since they almost certainly were not also taking an OC, they were likely excluded. We would expect these women to exhibit behaviours similar to women undergoing clomiphene treatment, but were unable to test this assumption.

## Conclusions

Peri-pregnancy patterns of prescription fills for analgesics and antibiotics did not vary substantially by the studied proxies for pregnancy planning. This suggests that there were few differences in medication use between women with planned and unplanned pregnancies, or that fertility treatment and timing of OC discontinuation from routinely collected health data, even when combined with data on folic acid use, cannot stand alone in the identification of unplanned pregnancies. A narrower definition of within-pregnancy OC discontinuation seemed a better proxy, but this should be confirmed in other studies. It should also be considered that women on fertility treatment or OC are not representative of all pregnancy planners. Future studies should examine how proxies for pregnancy intention perform, and whether pregnancies can be identified prospectively, in a cohort that is not selected based on achieving and maintaining a pregnancy beyond the 12th gestational week. To enable identification of pregnancy intention in a larger proportion of the population, future studies could consider more data-driven methods making use of a wider range of health indicators as proxies for pregnancy intention, such as age, smoking cessation, removal of long-acting hormonal contraceptives, and discontinuation of others medications.

## Supplementary Information

**Additional file 1.** Characteristics of the included pregnancies by proxies of pregnancy intention, stratified on folic acid use.

**Additional file 2.** Analgesic prescription fills by proxies of pregnancy intention, stratified on folic acid use. Proportion of pregnancies with analgesic prescription fills by peri-pregnancy period and proxies of pregnancy intention, stratified on folic acid use.

**Additional file 3.** Antibiotic prescription fills by proxies of pregnancy intention, stratified on folic acid use. Proportion of pregnancies with antibiotic prescription fills by peri-pregnancy period and proxies of pregnancy intention, stratified on folic acid use.

**Additional file 4.** Results from sensitivity analysis redefining pregnancy start as 14 days earlier. Characteristics of included pregnancies, proportion of pregnancies with analgesic prescription fills, proportion of pregnancies with antibiotic prescription fills by proxies of pregnancy intention, sensitivity analysis redefining pregnancy start as 14 days earlier.

**Additional file 5.** Results from sensitivity analysis redefining pregnancy start as 14 days later. Characteristics of included pregnancies, proportion of pregnancies with analgesic prescription fills, proportion of pregnancies with antibiotic prescription fills by proxies of pregnancy intention, sensitivity analysis redefining pregnancy start as 14 days later.

**Additional file 6.** Results from sensitivity analysis including women with filled prescriptions for other short-acting hormonal contraceptives. Characteristics of included pregnancies, proportion of pregnancies with analgesic prescription fills, proportion of pregnancies with antibiotic prescription fills by proxies of pregnancy intention, sensitivity analysis including women with filled prescriptions of vaginal rings with hormones and/or contraceptive patches.

**Additional file 7.** Results from sensitivity analysis restricting to live births. Characteristics of included pregnancies, proportion of pregnancies with analgesic prescription fills, proportion of pregnancies with antibiotic prescription fills by proxies of pregnancy intention, sensitivity analysis restricting to live births.

**Additional file 8.** Results from sensitivity analysis with a more narrow definition of oral contraceptive discontinuation during pregnancy. Characteristics of included pregnancies, proportion of pregnancies with analgesic prescription fills, proportion of pregnancies with antibiotic prescription fills by proxies of pregnancy intention, sensitivity analysis redefining oral contraceptive discontinuation during pregnancy as fillings within one cycle before pregnancy.

**Additional file 9.** Characteristics of pregnancies by inclusion and exclusion from the study sample.

## Data Availability

The data that support the findings of this study are available from the Norwegian Institute of Public Health but restrictions apply to the availability of these data, which were used under license for the current study, and so are not publicly available. Data are however available from the authors upon reasonable request and with permission of a Norwegian Regional Committee for Research Ethics, a Data Protection Officer at a Norwegian research institution, and the Norwegian Institute of Public Health.

## Abbreviations

ATC: Anatomical Therapeutic Chemical
MBRN: Medical Birth Registry of Norway
NorPD: Norwegian Prescription Database
OC: Oral contraceptives
