# Supplementary material for: Fertility treatment and oral contraceptive discontinuation for identification of pregnancy planning in routinely collected health data – an application to analgesic and antibiotic utilisation
Source: BMC Pregnancy Childbirth. 2020 Nov 25;20:731. doi: 10.1186/s12884-020-03435-4 (PMC7690077; doi:10.1186/s12884-020-03435-4)
Supplement: Supplementary file 1 — Additional file 1. Characteristics of the included pregnancies by proxies of pregnancy intention, stratified on folic acid use. [file 12884_2020_3435_MOESM1_ESM.docx]

**Additional file 1: Characteristics of the included pregnancies by proxies of pregnancy intention^a^.**

|  |  | | | Timing of oral contraceptive discontinuation | | | | | | | | |
| --- | --- | --- | --- | --- | --- | --- | --- | --- | --- | --- | --- | --- |
|  | Fertility treatment  (n=19 449) | | | Early  (n=77 735) | | | Late  (n=42 621) | | | Within-pregnancy  (n=32 780) | | |
|  | Folate before (n:9912) | Folate during (n:6101) | No folate (n:3436) | Folate before  (n:27416) | Folate during (n:33513) | No folate (n:16806) | Folate before (n:14008) | Folate during (n:19127) | No folate (n:9486) | Folate before (n:8324) | Folate during (n:16111) | No folate (n:8345) |
| Maternal age | 32.1 (4.8) | 31.1 (5.0) | 31.9 (5.2) | 29.7 (4.2) | 28.4 (4.7) | 28.6 (5.2) | 29.2 (4.1) | 27.9 (4.5) | 28.2 (5.0) | 28.8 (4.3) | 27.3 (4.8) | 27.4 (5.4) |
| Married/cohabiting | 95.9 | 95.4 | 93.6 | 97.3 | 93.6 | 91.4 | 97.4 | 94.4 | 92.3 | 96.4 | 92.1 | 88.9 |
| Employed | 79.9 | 76.2 | 48.7 | 82.3 | 77.3 | 54.7 | 82.8 | 78.6 | 58.6 | 80.2 | 75.6 | 57.1 |
| Nulliparous | 61.3 | 56.3 | 52.4 | 55.8 | 57.1 | 50.6 | 55.9 | 60.7 | 53.7 | 57.3 | 62.8 | 55.0 |
| Previous pregnancy loss | 29.5 | 26.4 | 23.5 | 24.6 | 17.7 | 16.6 | 12.2 | 10.2 | 9.7 | 9.8 | 9.9 | 9.9 |
| Obstetric comorbidity index^b^  *Components of the index*  Asthma  Diabetes, pre-gestational  Hypertension, chronic  Hypertension, gestational  Kidney disease  Multiple gestation  Preeclampsia, mild  Preeclampsia, severe  Previous caesarean section | 0.78 (1.2)  5.8  1.2  0.9  2.3  0.6  5.9  2.9  2.0  5.4 | 0.72 (1.2)  5.3  1.4  1.0  1.9  0.5  5.2  3.0  2.3  5.8 | 0.81 (1.2)  3.8  1.3  0.9  4.0  0.6  5.6  3.2  2.2  5.4 | 0.41 (0.9)  5.5  0.7  0.6  2.1  0.7  1.3  1.9  1.3  5.6 | 0.37 (0.8)  5.6  0.6  0.5  1.9  0.7  1.2  2.1  1.1  5.2 | 0.38 (0.9)  4.1  0.6  0.5  2.4  0.5  1.4  2.1  1.2  5.3 | 0.37 (0.8)  5.1  0.8  0.5  2.0  0.7  1.4  2.0  1.2  5.1 | 0.34 (0.8)  5.8  0.6  0.4  2.0  0.6  1.1  2.3  1.3  4.4 | 0.37 (0.9)  4.4  0.8  0.4  2.6  0.6  1.4  1.8  1.6  4.4 | 0.36 (0.9)  5.4  0.8  0.6  2.0  0.6  1.4  1.8  1.5  4.0 | 0.33 (0.9)  5.7  0.6  0.4  1.6  0.6  1.1  2.2  1.6  3.6 | 0.37 (0.9)  4.8  0.7  0.5  2.1  0.5  1.3  2.2  1.9  3.4 |
| Rheumatoid arthritis | 0.9 | 0.5 | 0.3 | 0.6 | 0.4 | 0.3 | 0.7 | 0.3 | 0.3 | 0.4 | 0.2 | 0.2 |
| Smoking in early pregnancy | 3.6 | 7.7 | 6.9 | 4.0 | 9.3 | 12.2 | 4.0 | 9.1 | 11.4 | 5.3 | 10.6 | 13.4 |
| Smoking at the end of pregnancy | 2.1 | 4.6 | 4.4 | 2.1 | 5.1 | 7.7 | 2.1 | 4.9 | 7.1 | 2.9 | 5.4 | 7.9 |
| Weight gain in pregnancy | 13.8 (7.7) | 13.9 (7.7) | 13.7 (7.9) | 14.2 (7.4) | 14.9 (9.3) | 14.3 (8.4) | 14.4 (7.1) | 14.9 (7.4) | 14.3 (7.7) | 14.6 (6.7) | 15.0 (7.9) | 15.0 (9.3) |

^a^Figures shown are percent of non-missing values with the exception of maternal age, calendar year, obstetric comorbidity index, and weight gain in pregnancy, presented as mean (standard deviation). Missing values ranged from 0% (maternal age, calendar year, parity) to 10.7% to 41.0% (maternal employment, with highest proportion of missing for women with no folic acid use). Women could choose not to have smoking and weight reported to the MBRN. For smoking, 7.7% to 36.3% chose not to report. For weight, 73.6% to 85.3% chose not to report.

^b^Adapted from Bateman et al. (22), using the variables available in MBRN (age, asthma, pre-gestational diabetes, chronic hypertension, kidney disease, previous caesarean section, multiple gestation, severe preeclampsia, mild preeclampsia, gestational hypertension) and weighting the variables as done by Bateman et al.
