# Supplementary material for: Fertility treatment and oral contraceptive discontinuation for identification of pregnancy planning in routinely collected health data – an application to analgesic and antibiotic utilisation
Source: BMC Pregnancy Childbirth. 2020 Nov 25;20:731. doi: 10.1186/s12884-020-03435-4 (PMC7690077; doi:10.1186/s12884-020-03435-4)
Supplement: Supplementary file 2 — Additional file 2. Analgesic prescription fills by proxies of pregnancy intention, stratified on folic acid use. Proportion of pregnancies with analgesic prescription fills by peri-pregnancy period and proxies of pregnancy intention, stratified on folic acid use. [file 12884_2020_3435_MOESM2_ESM.docx]

**Additional file 2: Analgesic prescription fills by proxies of pregnancy intention, stratified on folic acid use^a^.**

|  |  | | | Timing of oral contraceptive discontinuation | | | | | | | | |
| --- | --- | --- | --- | --- | --- | --- | --- | --- | --- | --- | --- | --- |
|  | Fertility treatment  (n=19 449) | | | Early  (n=77 735) | | | Late  (n=42 621) | | | Within-pregnancy  (n=32 780) | | |
|  | Folate before (n:9912) | Folate during (n:6101) | No folate (n:3436) | Folate before  (n:27416) | Folate during (n:33513) | No folate (n:16806) | Folate before (n:14008) | Folate during (n:19127) | No folate (n:9486) | Folate before (n:8324) | Folate during (n:16111) | No folate (n:8345) |
| *Any analgesics* | | | | | | | | | | | | |
| 3 months before pregnancy start | 10.9 | 11.4 | 13.0 | 9.2 | 9.3 | 9.6 | 8.8 | 9.7 | 9.7 | 9.9 | 10.6 | 11.0 |
| First trimester | 4.2 | 5.3 | 4.7 | 3.5 | 4.0 | 4.6 | 3.5 | 4.1 | 4.4 | 4.3 | 5.0 | 5.3 |
| Second trimester | 2.0 | 2.5 | 2.4 | 1.6 | 1.7 | 1.9 | 1.7 | 1.7 | 2.0 | 1.6 | 1.9 | 2.0 |
| Third trimester | 2.2 | 2.1 | 2.4 | 1.5 | 1.6 | 1.7 | 1.4 | 1.6 | 1.6 | 1.7 | 1.6 | 1.7 |
| 3 months after pregnancy end | 7.8 | 8.3 | 8.3 | 5.3 | 5.7 | 5.5 | 5.1 | 5.2 | 5.2 | 5.1 | 5.3 | 5.3 |
| *Non-steroidal anti-inflammatory drugs* | | | | | | | | | | | | |
| 3 months before pregnancy start | 6.1 | 7.1 | 8.3 | 5.3 | 5.9 | 6.2 | 5.5 | 6.3 | 6.1 | 6.3 | 7.1 | 7.2 |
| First trimester | 1.3 | 2.1 | 2.2 | 1.5 | 2.0 | 2.3 | 1.5 | 2.1 | 2.1 | 2.0 | 2.5 | 2.9 |
| Second trimester | 0.2 | 0.3 | 0.4 | 0.2 | 0.2 | 0.3 | 0.2 | 0.2 | 0.3 | 0.2 | 0.2 | 0.4 |
| Third trimester | 0.1 | 0.2 | 0.3 | 0.1 | 0.1 | 0.1 | 0.1 | 0.1 | 0.1 | 0.2 | 0.2 | 0.2 |
| 3 months after pregnancy end | 5.1 | 5.4 | 4.8 | 3.5 | 3.8 | 3.6 | 3.3 | 3.6 | 3.4 | 3.4 | 3.4 | 3.5 |

^a^Proportion of pregnancies with filled prescriptions for analgesics, %
