# Supplementary material for: Fertility treatment and oral contraceptive discontinuation for identification of pregnancy planning in routinely collected health data – an application to analgesic and antibiotic utilisation
Source: BMC Pregnancy Childbirth. 2020 Nov 25;20:731. doi: 10.1186/s12884-020-03435-4 (PMC7690077; doi:10.1186/s12884-020-03435-4)
Supplement: Supplementary file 3 — Additional file 3. Antibiotic prescription fills by proxies of pregnancy intention, stratified on folic acid use. Proportion of pregnancies with antibiotic prescription fills by peri-pregnancy period and proxies of pregnancy intention, stratified on folic acid use. [file 12884_2020_3435_MOESM3_ESM.docx]

**Additional file 3: Antibiotic prescription fills by proxies of pregnancy intention, stratified on folic acid use^a^.**

|  |  | | | Timing of oral contraceptive discontinuation | | | | | | | | |
| --- | --- | --- | --- | --- | --- | --- | --- | --- | --- | --- | --- | --- |
|  | Fertility treatment  (n=19 449) | | | Early  (n=77 735) | | | Late  (n=42 621) | | | Within-pregnancy  (n=32 780) | | |
|  | Folate before (n:9912) | Folate during (n:6101) | No folate (n:3436) | Folate before  (n:27416) | Folate during (n:33513) | No folate (n:16806) | Folate before (n:14008) | Folate during (n:19127) | No folate (n:9486) | Folate before (n:8324) | Folate during (n:16111) | No folate (n:8345) |
| *Any antibiotics* | | | | | | | | | | | | |
| 3 months before pregnancy start | 10.9 | 11.9 | 12.3 | 10.9 | 11.7 | 12.3 | 11.4 | 12.2 | 12.2 | 12.5 | 13.9 | 14.3 |
| First trimester | 9.8 | 11.9 | 13.0 | 10.1 | 11.3 | 12.2 | 10.1 | 11.8 | 12.4 | 11.3 | 13.4 | 13.3 |
| Second trimester | 12.1 | 13.0 | 13.3 | 11.0 | 12.2 | 13.0 | 10.7 | 12.8 | 13.2 | 11.4 | 13.1 | 14.3 |
| Third trimester | 13.6 | 14.5 | 14.6 | 12.0 | 13.9 | 13.9 | 12.4 | 13.7 | 14.3 | 13.1 | 14.1 | 15.7 |
| 3 months after pregnancy end | 20.3 | 21.4 | 20.9 | 17.8 | 18.3 | 17.9 | 17.8 | 18.4 | 17.6 | 18.4 | 18.2 | 17.7 |
| *Tetracyclines* | | | | | | | | | | | | |
| 3 months before pregnancy start | 1.4 | 1.5 | 1.4 | 1.1 | 1.4 | 1.3 | 1.3 | 1.3 | 1.4 | 1.4 | 1.7 | 1.7 |
| First trimester | 0.7 | 0.6 | 0.8 | 0.5 | 0.7 | 0.7 | 0.4 | 0.7 | 0.6 | 0.5 | 0.8 | 0.7 |
| Second trimester^b^ | <5 | <5 | 0 | 8 | 14 | <5 | <5 | 10 | 7 | <5 | 10 | 8 |
| Third trimester^b^ | <5 | <5 | 0 | <5 | 5 | <5 | <5 | <5 | 0 | 0 | <5 | <5 |
| 3 months after pregnancy end | 0.2 | 0.2 | 0.3 | 0.2 | 0.2 | 0.3 | 0.2 | 0.3 | 0.3 | 0.2 | 0.3 | 0.4 |

^a^Proportion of pregnancies with filled prescriptions for antibiotics, %

^b^Results not shown for reasons of confidentiality, when less than five pregnancies were exposed in a group.
