# Supplementary material for: Fertility treatment and oral contraceptive discontinuation for identification of pregnancy planning in routinely collected health data – an application to analgesic and antibiotic utilisation
Source: BMC Pregnancy Childbirth. 2020 Nov 25;20:731. doi: 10.1186/s12884-020-03435-4 (PMC7690077; doi:10.1186/s12884-020-03435-4)
Supplement: Supplementary file 5 — Additional file 5. Results from sensitivity analysis redefining pregnancy start as 14 days later. Characteristics of included pregnancies, proportion of pregnancies with analgesic prescription fills, proportion of pregnancies with antibiotic prescription fills by proxies of pregnancy intention, sensitivity analysis redefining pregnancy start as 14 days later. [file 12884_2020_3435_MOESM5_ESM.docx]

**Additional file 5: Results from sensitivity analysis redefining pregnancy start as 14 days later.**

Characteristics of the included pregnancies by proxies of pregnancy intention, start of pregnancy 14 days later than in the main analysis^a^.

|  |  | | | Timing of oral contraceptive discontinuation | | | | | | | | |
| --- | --- | --- | --- | --- | --- | --- | --- | --- | --- | --- | --- | --- |
|  | Fertility treatment  (n=20 353) | | | Early  (n=82 640) | | | Late  (n=44 049) | | | Within-pregnancy  (n=25 803) | | |
|  | Folate before (n:10353) | Folate during (n:6395) | No folate (n:3605) | Folate before  (n:29125) | Folate during (n:35655) | No folate (n:17860) | Folate before (n:14090) | Folate during (n:20001) | No folate (n:9958) | Folate before (n:6193) | Folate during (n:12834) | No folate (n:6776) |
| Maternal age | 32.1 (4.8) | 31.0 (5.0) | 31.9 (5.2) | 29.7 (4.2) | 28.4 (4.7) | 28.6 (5.2) | 29.1 (4.1) | 27.9 (4.5) | 28.1 (5.1) | 28.8 (4.3) | 27.2 (4.8) | 27.3 (5.4) |
| Married/cohabiting | 95.9 | 95.4 | 93.6 | 97.3 | 93.7 | 91.5 | 97.5 | 94.4 | 91.9 | 96.1 | 91.7 | 88.3 |
| Employed | 79.7 | 76.3 | 48.6 | 82.2 | 77.4 | 55.0 | 82.6 | 78.4 | 58.5 | 80.1 | 75.1 | 56.6 |
| Nulliparous | 60.5 | 55.9 | 51.9 | 55.5 | 57.2 | 50.6 | 55.4 | 60.3 | 53.5 | 57.3 | 62.7 | 55.2 |
| Previous pregnancy loss | 29.4 | 26.5 | 23.5 | 23.7 | 17.2 | 16.2 | 11.6 | 9.9 | 9.6 | 9.8 | 10.0 | 10.1 |
| Obstetric comorbidity index^b^  *Components of the index*  Asthma  Diabetes, pre-gestational  Hypertension, chronic  Hypertension, gestational  Kidney disease  Multiple gestation  Preeclampsia, mild  Preeclampsia, severe  Previous caesarean section | 0.77 (1.2)  5.6  1.2  0.9  2.3  0.6  6.0  2.9  1.9  5.5 | 0.72 (1.2)  5.3  1.3  1.0  1.9  0.5  5.3  3.2  2.2  6.0 | 0.80 (1.2)  3.7  1.2  0.8  3.9  0.6  5.7  3.1  2.1  5.4 | 0.41 (0.9)  5.5  0.8  0.5  2.1  0.7  1.2  1.9  1.2  5.6 | 0.37 (0.8)  5.6  0.6  0.5  1.9  0.8  1.2  2.1  1.1  5.2 | 0.38 (0.9)  4.2  0.6  0.5  2.4  0.5  1.4  2.1  1.3  5.2 | 0.36 (0.8)  5.2  0.8  0.5  2.0  0.7  1.4  1.8  1.2  5.0 | 0.34 (0.8)  5.7  0.6  0.4  1.9  0.6  1.1  2.2  1.3  4.2 | 0.38 (0.9)  4.6  0.8  0.4  2.5  0.6  1.6  1.8  1.7  4.3 | 0.37 (0.9)  5.4  0.8  0.6  1.9  0.6  1.5  2.1  1.6  3.9 | 0.34 (0.9)  5.6  0.6  0.4  1.7  0.6  1.1  2.2  1.6  3.7 | 0.37 (0.9)  4.8  0.7  0.5  2.1  0.6  1.1  2.3  1.9  3.4 |
| Rheumatoid arthritis | 0.9 | 0.5 | 0.3 | 0.6 | 0.4 | 0.3 | 0.6 | 0.3 | 0.3 | 0.5 | 0.2 | 0.2 |
| Smoking in early pregnancy | 3.5 | 7.5 | 6.8 | 4.0 | 9.2 | 12.1 | 4.1 | 9.1 | 11.5 | 5.6 | 10.8 | 14.0 |
| Smoking at the end of pregnancy | 2.0 | 4.5 | 4.4 | 2.2 | 4.9 | 7.8 | 2.1 | 5.0 | 7.0 | 3.1 | 5.6 | 8.1 |
| Weight gain in pregnancy | 13.8 (7.8) | 13.9 (7.6) | 13.7 (7.9) | 14.2 (7.3) | 14.9 (9.1) | 14.3 (8.7) | 14.4 (6.7) | 15.0 (7.6) | 14.2 (7.5) | 14.5 (7.0) | 15.0 (8.0) | 15.0 (8.9) |

^a^Figures shown are percent of non-missing values with the exception of maternal age, calendar year, obstetric comorbidity index, and weight gain in pregnancy, presented as mean (standard deviation). Missing values ranged from 0% (maternal age, calendar year, parity) to 10.7% to 40.9% (maternal employment, with highest proportion of missing for women with no folic acid use). Women could choose not to have smoking and weight reported to the MBRN. For smoking, 7.8% to 36.8% chose not to report. For weight, 73.8% to 85.7% chose not to report.

^b^Adapted from Bateman et al. (22), using the variables available in MBRN (age, asthma, pre-gestational diabetes, chronic hypertension, kidney disease, previous caesarean section, multiple gestation, severe preeclampsia, mild preeclampsia, gestational hypertension) and weighting the variables as done by Bateman et al.

**Proportion of pregnancies with filled prescriptions for analgesics by peri-pregnancy period and proxies of pregnancy intention, start of pregnancy defined as 14 days later than in the main analysis.** T: Trimester.

**Proportion of pregnancies with filled prescriptions for antibiotics by peri-pregnancy period and proxies of pregnancy intention, start of pregnancy defined as 14 days later than in the main analysis.** T: Trimester.
